# A Mixed-Method Application of the Program Sustainability Assessment Tool to Evaluate the Sustainability of 4 Pediatric Asthma Care Coordination Programs

**DOI:** 10.5888/pcd12.150133

**Published:** 2015-12-03

**Authors:** Shelley Stoll, Mary Janevic, Marielena Lara, Gilberto Ramos-Valencia, Tyra Bryant Stephens, Victoria Persky, Kimberly Uyeda, Yvonne Ohadike, Floyd Malveaux

**Affiliations:** Author Affiliations: Mary Janevic, University of Michigan School of Public Health, Ann Arbor, Michigan; Marielena Lara, RAND Corporation, Santa Monica, California; Gilberto Ramos-Valencia, University of Puerto Rico School of Public Health, San Juan, Puerto Rico; Tyra Bryant Stephens, Children’s Hospital of Philadelphia, Philadelphia, Pennsylvania; Victoria Persky, University of Illinois at Chicago School of Public Health, Chicago, Illinois; Kimberly Uyeda, Los Angeles Unified School District, Los Angeles, California; Yvonne Ohadike, Floyd Malveaux, Merck Childhood Asthma Network, Washington, DC.

## Abstract

**Introduction:**

As part of a cross-site evaluation of the implementation of an evidence-based intervention for pediatric asthma care coordination into low-income communities, we sought to understand the factors that influenced the programs’ expected sustainability of the programs after external funding ended.

**Methods:**

We administered the Center for Public Health Systems Science’s Program Sustainability Assessment Tool, a 40-item instrument assessing 8 domains of sustainability capacity, to 12 key informants across 4 program sites. We developed open-ended probes for each domain. We examined patterns in site-specific and overall domain scores, and coded qualitative data to identify challenges and strategies in each domain.

**Results:**

Across sites, the domains of program evaluation (cross-site mean, 5.4 on a scale of 1–7) and program adaptation (mean, 5.2) had the highest ratings (indicating a strong finding during program evaluation) and funding stability had the lowest rating (mean, 2.7). Scores varied most across sites in the domains of strategic planning (SD, 0.9) and funding stability (SD, 0.9). Qualitative data revealed key challenges, including how implementation difficulties and externally led implementation can impede planning for sustainability. Program leaders discussed multiple strategies for enhancing capacity within each domain, including capitalizing on the interconnectedness of all domains, such as using evaluation and communication strategies to bolster internal political support throughout the implementation process.

**Conclusion:**

Findings indicating weak and strong domains were consistent with previous findings of studies that used the Program Sustainability Assessment Tool. The addition of qualitative probes yielded detailed data describing capacity strengths, weaknesses, and strategies to increase the likelihood that programs are sustained.

## Introduction

Understanding how effective health programs can be sustained over time is of interest to researchers, practitioners, and funders (1,2). Sustainability is the final phase in a program’s life cycle, in which research-based interventions are integrated and institutionalized within local contexts, often after external funding is terminated (1,3,4). Program sustainability is a complex, dynamic phenomenon (5,6) and the factors that are necessary and sufficient to ensure sustainability for different intervention types and in diverse contexts have not been fully explicated (1,6,7).

The literature on sustainability is fragmented (7), and most instruments to assess sustainability were developed for specific projects (8). Using established frameworks and validated tools appropriate for a broad range of programs may help build an evidence base that moves the field forward. As part of a cross-site evaluation of the implementation of an evidence-based intervention for pediatric asthma care coordination, we sought to understand the factors influencing the expected sustainability of an evidence-based intervention in 4 community settings: a neighborhood, a school system, a clinic, and a health care system (9). Data collection was guided by the Program Sustainability Assessment Tool (PSAT), a 40-item multiple-choice instrument that assesses a program’s sustainability capacity in 8 domains (10). We chose the PSAT over other frameworks because its developers created and tested a corresponding easy-to-use, reliable tool that is not program-specific (11–13) but rather is applicable to various public health programs (10). Following the recommendation by Wiltsey Stirman et al (2) that sustainability research include qualitative aspects, we developed open-ended probes to supplement close-ended items in each domain. Our primary research questions were the following: In a grant-funded, multisite implementation of a pediatric asthma care-coordination evidence-based intervention, how was sustainability capacity across 8 domains rated by site leaders and staff? What do site leaders identify as successful strategies and biggest challenges for bolstering sustainability capacity within each domain? Secondarily, we sought to explore the strengths and limitations of the PSAT as a tool for evaluation.

## Methods

The Merck Childhood Asthma Network (MCAN) funded 4 sites from 2010 to 2014 to demonstrate the effectiveness of care coordination in communities with significant disparities in asthma illness and death. This funding was for the second phase of a 2-part initiative to improve pediatric asthma care and outcomes in underserved communities (14–16). All 4 sites adapted the evidence-based intervention *Yes We Can* to fit each site’s priority population, organizational context, and leadership structure. *Yes We Can* is a medical-social model of care that provides asthma education, links families to health and social services, and facilitates patient–provider communication (17–19). The 4 program sites (Table 1) had different key characteristics. In Chicago (neighborhood site), academic researchers partnered with clinical and community organizations to address asthma at the neighborhood level. In Philadelphia (health system site), a large pediatric health system focused on patients at primary care clinics that serve inner-city families. In San Juan, Puerto Rico (federally qualified health center [FQHC] site), academics teamed with an FQHC to reach the center’s patients. In Los Angeles (school site), nursing services in a large public school district partnered with Breathmobiles to reach students with poor asthma control.

**Table 1:** Setting, Priority Population, Lead Implementers, and Key Partners of 4 Program Sites in the Merck Childhood Asthma Network

Setting	Priority Population	Lead Implementers	Key Partners
Neighborhood	Residents of Englewood neighborhood	University of Illinois at Chicago School of Public Health	• Damen Clinic • Beloved Clinic (FQHC) • St. Bernard’s Hospital • Teamwork Englewood
Health system	Patients of Children’s Hospital of Philadelphia’s inner-city primary care practices	The Community Asthma Prevention Program, Children’s Hospital of Philadelphia	• Primary Care Centers (physicians and staff; ED^a^ and inpatient records) • Asthma champions^b^
Federally Qualified Health Center (FQHC)	Patients of HealthproMed (FQHC)	University of Puerto Rico School of Public Health in partnership with RAND Health	• HealthproMed, Inc (FQHC) • Community leaders and organization in catchment area of FQHC
School	Students of Los Angeles Unified School District	Los Angeles Unified School District Division of Student and Health and Human Services	• The Los Angeles Unified School District, School Nurses^b^ • LA County and USC Breathmobile Clinic^c^ • School health clinics

a Emergency department.

b Partner is part of the same institution as the lead implementer.

c Los Angeles and University of Southern California Breathmobile Clinic.

### Measures

Version 1 of the PSAT assesses 8 domains of sustainability capacity (political support, funding stability, partnerships, organizational capacity, program evaluation, program adaptation, communications, and strategic planning) with 5 items each. Respondents rate the extent (1, little or no extent to 7, a very great extent) to which the program has or does what the item describes (eg, “Diverse community organizations are invested in the success of the program”). PSAT subscales demonstrated high internal consistency and reliability in a sample of 592 respondents representing 252 public health programs (10). Because the tool’s brevity and simplicity may limit its ability to capture nuances in local settings (20), the authors encourage program teams to discuss domains as a whole. To address this limitation when using the tool for evaluation, we developed open-ended questions for each domain (eg, “Describe the strengths and weaknesses you see in terms of the organizational capacity to maintain this program”). (See online Appendix for PSAT tool with qualitative probes.)

In 2014, we conducted 12 key informant interviews (2–4 per-site; average length, 80 minutes; 100% response rate) by telephone with each site’s principal investigators, program managers, and (at one site) the medical director of a partner clinic. Before the interview, the respondent was emailed the PSAT and asked to 1) complete the close-ended items and 2) think about answers to the open-ended questions. In audio-recorded interviews, respondents first reported their responses to the PSAT items and then answered the open-ended questions. A research assistant listened to all audio recordings to add details to interviewer notes and capture illustrative quotations.

### Data coding and analysis

We calculated site-specific means for each of the 40 items. Site-specific domain scores were obtained by averaging item scores within a domain. Overall domain scores were obtained by averaging the 4 site scores for each domain, and standard deviations were calculated to show variability by site. We also calculated cross-site average scores for each item.

To develop a coding scheme, notes from 3 randomly selected interviews were each independently coded by 2 evaluators. A priori codes were derived from PSAT items, with subcodes indicating strategies and challenges. Other codes emerged from data, and evaluators came to consensus on a codebook. Two researchers independently coded the remaining notes, compared codes and came to consensus, modifying the codebook as needed to ensure convergence and divergence of the coding scheme (13).

## Results

### PSAT domain scores

Each sustainability capacity domain is defined, and mean domain scores across sites are presented (Table 2). The domains of program evaluation (mean, 5.4) and program adaptation (mean = 5.2) had the highest ratings; funding stability (mean, 2.7), political support (mean, 4.1), and strategic planning (mean, 4.2) had the lowest (Figure). Site-specific domain scores ranged from a low of 1.4 (funding stability, neighborhood site) to a high of 5.8 (strategic planning, school site). Across domains, the school site had the highest scores, followed by the health system site, neighborhood site, and FQHC site. Scores for strategic planning and funding stability varied most by site (for both, SD = 0.9).

**Table 2:** PSAT^a^ Domains, Definitions, Cross-Site Means, and Standard Deviations of 4 Program Sites in the Merck Childhood Asthma Network, 2014

Domain	Definition	Cross-Site Mean Score^b^ (SD)
Program evaluation	Assessing your program to inform planning and document results	5.4 (0.1)
Program adaptation	Taking actions that adapt your program to ensure its ongoing effectiveness	5.2 (0.1)
Organization capacity	Having the internal support and resources needed to effectively manage your program	5.0 (0.1)
Communications	Strategic communication with stakeholders and the public about your program	4.9 (0.5)
Partnerships	Cultivating connections between your program and its stakeholder	4.5 (0.2)
Strategic planning	Using processes that guide your program’s directions, goals, and strategies	4.2 (0.9)
Political support	Having a supportive internal and external climate for your program	4.1 (0.7)
Funding stability	Establishing a consistent financial base for your program.	2.7 (0.9)

a Program Sustainability Assessment Tool (8).

b Possible range: 1–7, with a higher number indicating greater strength in the domain.

**Figure:**
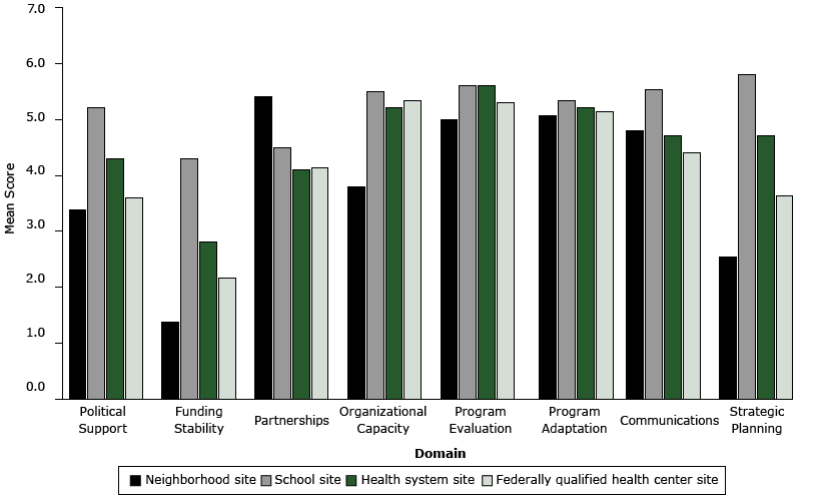
Mean PSAT Sustainability Capacity Scores by Domain and Site in the Merck Childhood Asthma Network. Possible scores range from 1 to 7, with a higher number indicating greater strength. Scores were determined based on assessments of site leaders (2–4 per site) of 5 items per domain. Abbreviation: FQHC, federally qualified health center. **Table:** 

Program Domain	Neighborhood Site	School Site	Health Care System Site	FQHC Site
Political support	3.3	5.2	4.3	3.6
Funding stability	1.4	4.3	2.8	2.2
Partnerships	5.4	4.5	4.1	4.1
Organizational capacity	3.8	5.5	5.2	5.3
Program evaluation	5.0	5.6	5.6	5.3
Program adaptation	5.1	5.3	5.2	5.1
Communications	4.8	5.5	4.7	4.4
Strategic planning	2.5	5.8	4.7	3.6

Open-ended responses provided insights into domain ratings. These are presented below in descending order of average domain score.

**Program evaluation (cross-site mean, 5.4, SD = 0.1).** Respondents indicated that they used evaluation findings to strengthen the program and its sustainability by tailoring messages for families, continuously improving the program, and generating and maintaining support. As one respondent put it, “People have to see a value in what you are doing before they completely invest in it.” Evaluations were strengthened by allocating sufficient resources, tapping into existing systems for data collection, and seeking regular input from staff and other stakeholders. Conversely, a few respondents reported that lack of access to health record data and inadequate process evaluation limited the effectiveness of evaluation on sustainability.

**Program adaptation (cross-site mean, 5.2, SD = 0.1).** Adaptability was seen as critical to program success and sustainability, and no respondents cited barriers to program adaptation. Respondents cited several reasons for adaptations: to adjust to shifting political environments; to work effectively with key partners; to boost participant engagement; to accommodate clinical workflows; and to leverage resources. One respondent emphasized that adapting the program to be more cost-effective promoted sustainability by “showing stakeholders that the program is in a position to adjust to the financial reality they live in.”

**Organizational capacity (cross-site mean, 5.0, SD = 0.1).** Respondents cited numerous organizational strengths promoting implementation and sustainability, including ongoing asthma quality improvement initiatives, ability to integrate coordinators into clinical workflow and staff meetings, coordinator access to electronic health records, a vast network of school nurses serving as a referral system, and support from key staff and board members. Respondents from all programs cited the ongoing challenge of organizations’ multiple competing priorities. Other commonly cited challenges were a lack of physical space, staffing, and extant referral systems.

**Communications (cross-site mean, 4.9, SD = 0.5).** Communication was seen as enhancing sustainability primarily by enhancing political and financial support for the program. Effective strategies were using channels such as the organization’s communications office, staff in-services, and relationships with community leaders; tailoring messages to the audience; and emphasizing positive evaluation results and the program’s immediate benefit to families. One respondent described how communication efforts garnered national recognition for the organization, increasing internal political support to continue the program. Challenges were insufficient skills, resources, and channels for communication.

**Partnerships (cross-site mean, 4.5, SD = 0.2).** Partner organizations and community members were viewed as contributing to sustainability by facilitating successful program implementation and offering political support. For example, the endorsement of community partners who act as gatekeepers to community members was critical to successful implementation at one site. Partnerships are especially critical for care coordination work, which seeks to connect families to resources such as housing assistance and smoking cessation. As one program manager said, “We’re not trying to do it all ourselves. We know our program limitations and we know our strengths, so if there is a partner that can help us, we definitely will tap into that.” Responses highlighted that partnerships are instrumental in generating political support to continue the work. For instance, FQHC board members successfully advocated for program components to continue beyond grant funding. Challenges were overcoming partner distrust, insufficient resources for a strong community advisory board, and low visibility in the community resulting from weak communication.

**Strategic planning (cross-site mean, 4.2, SD = 0.9).** Two sites invested considerable resources in strategic planning for sustainability. In the health system site, respondents noted that having sustainability in mind from the outset was key to their success. The program was designed so that the care coordinators were housed in-clinic and viewed as part of the health care team. This approach made it easier to make the coordinators’ staff positions permanent eventually and to have a major payer agree to cover a home visit for children with poor asthma control. In contrast, another site encountered so many implementation barriers that it switched its primary clinical partner midway through the funding period, and the demands of implementation precluded a focus on sustainability planning.

**Political support (cross-site mean, 4.1, SD = 0.7).** Among the challenges to political support for sustainability were local government elections that resulted in the loss of powerful advocates; lack of policy incentives; community economic hardship; organizational disarray and high turnover; lack of a local external group focused on awareness and advocacy for asthma; and the challenge of garnering support for a health program within a school district. A few respondents referred to the lack of buy-in from clinicians who are overwhelmed with responsibilities, lack interest in the program, or both.

Respondents described strategies that strengthened political support: demonstrating return on investment to organizational leaders; creating powerful champions by involving organizational leaders from the beginning; and tying the program to the organization’s core mission. For example, the school site emphasized the program’s impact on reducing school absences, and program support was bolstered when the local district attorney called for an end to asthma-related absences. Others built support by providing reimbursable services that generated revenue. Strategies for garnering clinician support were reducing their workloads, aligning incentives, and engaging program champions.

Several respondents mentioned characteristics of asthma itself that facilitated political support: its high prevalence and its status as the most common reason for pediatric emergency department visits, significant disparities in asthma outcomes, asthma not stigmatized like sexually transmitted infections, and the relative ease with which positive outcomes can be produced, in contrast to conditions such as obesity.

**Funding stability (cross-site mean, 2.7, SD = 0.9).** Maintaining funding stability was difficult for all sites. Most respondents cited challenges stemming from the larger economic and political context (eg, impoverished communities with weak infrastructure and few influxes of funding, a local health insurance system that will not reimburse for care coordination services, defunding of health services, and fewer grant opportunities). Some reported strengthening funding stability by seeking in-kind support and cost sharing, pursuing multiple avenues of support, and being flexible about which program components are supported.

## Discussion

As part of a broader evaluation of pediatric asthma care coordination interventions in 4 urban communities, we used the PSAT to evaluate sustainability capacity in 8 domains, adding our own open-ended probes. The relative strengths of the 8 sustainability capacity domain scores were fairly consistent across sites. The domains of program evaluation and program adaptation were rated highly at all sites. The funder required sites to have a robust evaluation component and provided resources for this expense. Similarly, the funder encouraged the sites to adapt the programs to optimize accessibility and impact of their services for hard-to-reach populations. The domains with the lowest scores across sites — funding stability, political support, and strategic planning — were also the most variable. The single item with the lowest average cross-site rating (2.4, “The program is funded through a variety of sources”) suggested a lack of diversification in funding streams across settings. (Cross-site item averages are not shown in the Table.) Funding stability is a common challenge described in the literature; Scheirer (21), for example, found that difficulty obtaining external or internal funding for program continuation was the most frequently reported barrier to sustainability in 48 projects with short-term foundation funding. Political support scores were also low, but our qualitative data indicate that some respondents assumed a narrow definition of “political” that pertained only to elected officials. The tool developers also identified this issue, and the domain name is now called environmental support (13).

Scores for the strategic planning domain — as well as overall site scores — were significantly lower in the 2 sites where program implementation was led by academic researchers in partnership with one or more organizations, compared with the 2 sites with programs that were led and housed internally. When lead implementers are internal to an organization, they may be better positioned to garner political and financial support. External implementers may encounter more barriers to strategic planning, and academic researchers in particular may be less able to direct resources toward sustainability. The discrepancies in scores for the item “The program plans for future resource needs” indicate that less planning took place in the 2 programs led by academic researchers (mean, 3.5) than in the 2 internally led programs (mean, 5.5).

Our findings mirror those from a previous assessment of 252 public health programs (10) that also found program adaptation and program evaluation to be highly rated and funding stability to be the lowest-rated domain. In another study that incorporated the PSAT, funding stability was the lowest-scoring domain (22). Because the PSAT is a fairly new tool, published applications are limited; the other studies that used or cited the PSAT do not report empirical data across domains (13,23,24).

Our qualitative findings highlighted the interconnectedness of domains; these findings, along with those from a review of empirical literature on sustainability (2), support conceptual models of interactive relationships among influences on sustainability (25). Implementers may capitalize on their program’s stronger capacities to boost other domains. For example, the neighborhood program may leverage its partnerships’ strengths through strategic planning sessions with partners that bring the perspectives and resources of diverse stakeholders to the table. Programs with strong evaluation components could present tailored evaluation findings to garner more political support. Qualitative findings also revealed leaders’ focus on securing continued funding for key implementing staff, supporting Scheirer’s hypothesis that sustainability of interventions requiring coordination among multiple staff members is strongly influenced by the availability of continued financial resources to support these staff positions (1).

Findings demonstrated the usefulness of the PSAT for guiding a mixed-methods evaluation of sustainability capacity. Although developers used open-ended probes during PSAT tool development, the qualitative questions we added were useful for evaluation purposes. Besides providing rich data about sustainability capacity, these questions also gave insight into how close-ended items were interpreted by respondents. This finding provides additional information on the validity of scale items, as called for by the scale developers (10).

First, all data were self-reported by a small number of individuals at each site. Self-report assessments can be inaccurate (26), and these individuals did not represent all stakeholders. If we had interviewed other stakeholders, such as staff from key partners, organizational leaders who oversee program leaders, and community representatives, additional strengths and weaknesses may have been revealed in each domain. Such stakeholders may have perspectives that differ from those immersed in the work, and they may be less likely to emphasize positive aspects. Third, our data represent a period before grant funding had ended. Ideally, research on sustainability is conducted at multiple time points (2), both before and after the end of funding, although, as in this case, evaluation funding ends with the grant (21). Although we used the PSAT in primarily a descriptive manner, our results suggest that the PSAT could be used prescriptively as well, to identify weaker areas and try to remedy them. Finally, findings may be most relevant to other interventions that require coordination among multiple staff members, because sustainability influences may vary depending on the type of intervention (1).

Despite the requirement by many funders that an implementing organization be able to maintain a successful program, this goal seems elusive in practice. Study findings can help inform community, public health, and health system programs throughout a program’s life cycle. In particular, the area of funding stability warrants special attention, as it continues to be the main barrier to sustainability. From the outset, program developers can deliberately design interventions that are likely to strengthen funding stability and the other domains that are consistently weak across studies. Likewise, funders, purveyors, and practitioners can promote structures and processes that strengthen factors in low-scoring domains, and all can harness the interconnectedness of the domains to leverage strong areas to boost those that are weak. Evaluators and researchers can use the PSAT to assess both program design and the strategies to promote sustainability used during the implementation process to continue to identify links between these and long-term program sustainment.

## Appendix

Program Sustainability Assessment Tool (PSAT) with qualitative probes

This file is available for download as a Microsoft Word document.
